# Effects of curtailed sleep on cardiac stress biomarkers following high-intensity exercise

**DOI:** 10.1016/j.molmet.2022.101445

**Published:** 2022-01-26

**Authors:** Teemu Martikainen, Fjola Sigurdardottir, Christian Benedict, Torbjørn Omland, Jonathan Cedernaes

**Affiliations:** 1 Department of Medical Sciences, Uppsala University, Sweden; 2 Department of Medical Cell Biology, Uppsala University, Sweden; 3 Department of Cardiology, Akershus University Hospital, Lørenskog, Norway; 4 Institute of Clinical Medicine, University of Oslo, Oslo, Norway; 5 Department of Surgical Sciences (Sleep Science Laboratory, BMC), Uppsala University, Sweden

**Keywords:** High-intensity exercise, Cardiomyocyte, Cardiovascular strain, Heart muscle, Sleep loss, Troponin, NT-proBNP, N-terminal pro-B-type natriuretic peptide, cTnI, cardiac troponin-I, cTnT, cardiac troponin-T

## Abstract

**Objective:**

Physical exercise—especially at high intensity—is known to impose cardiac stress, as mirrored by, e.g., increased blood levels of cardiac stress biomarkers such as cardiac Troponin T (cTnT) and NT-proBNP. We examined healthy young participants to determine whether a few nights of short sleep duration alter the effects of acute exercise on these blood biomarkers.

**Methods:**

Sixteen men participated in a randomized order in a crossover design, comprising three consecutive nights of a) normal sleep duration (NS, 8.5 h of sleep/night) and b) sleep restriction (SR, 4.25 h of sleep/night). Blood was repeatedly sampled for determination of NT-proBNP and cTnT serum levels before and after a high-intensity exercise protocol (i.e., 75% VO_2_^*maxReserve*^ cycling on an ergometer).

**Results:**

Under pre-exercise sedentary conditions, blood levels of cTnT and NT-proBNP did not significantly differ between the sleep conditions (P > 0.10). However, in response to exercise, the surge of circulating cTnT was significantly greater following SR than NS (+37–38% at 120–240 min post-exercise, P ≤ 0.05). While blood levels of NT-proBNP rose significantly in response to exercise, they did not differ between the sleep conditions.

**Conclusion:**

Recurrent sleep restriction may increase the cardiac stress response to acute high-intensity exercise in healthy young individuals. However, our findings must be further confirmed in women, older subjects and in patients with a history of heart disease.

## Highlights

• Chronic sleep curtailment increases the risk of cardiovascular disease.
• Here, we examined whether exercise-induced cardiac strain in healthy young adults is altered by sleep curtailment.
• Blood levels of the cardiac stress marker troponin were higher after exercise under conditions of recurrent sleep restriction.
• Sleep restriction may increase exercise-induced cardiac strain in adults.

## Graphical abstract

**Figure:**
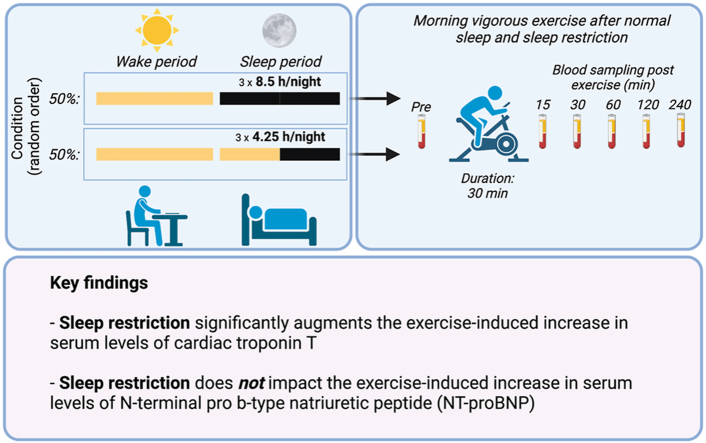

## 1 Introduction

Several blood biomarkers are routinely used in the clinic to assess heart damage and cardiovascular workload, such as high-sensitivity cardiac-specific troponin (hs-cTnT/cTnI) and natriuretic peptides (such as N-terminal pro-B-type natriuretic peptide; NT-proBNP). While some studies suggest that circulating levels of these factors exhibit diurnal (sleep/wake) or true circadian rhythms in humans [[1], [2], [3], [4]], we currently lack knowledge about the extent to which acute periods of sleep loss *per se* may alter the circulating levels of hs-cTnT and NT-proBNP. Importantly, such changes may primarily be seen in response to the addition of another physiologic stressor, such as acute exercise.

Physical exercise—especially at high intensity—is known to impose stress on the cardiovascular system. Regular exercise at a vigorous intensity and/or extensive duration can promote atherosclerotic changes and subclinical myocardial damage [5,6]. Indeed, acute exercise has consistently been found to increase circulating levels of cTnT/TnI and NT-proBNP [7,8]. Such release may represent physiological processes due to the cardiovascular stress of exercise.

Here we used highly standardized within-subject conditions to study healthy, normal weight men after three nights of short sleep duration and following three nights of normal sleep. By having participants carry out an acute bout of vigorous exercise, we tested whether circulating levels of hs-cTnT and NT-proBNP are altered by sleep loss *per se,* or whether this occurs only when additional cardiovascular stress is imposed.

## 2 Material and methods

### 2.1 Participants and screening procedure

For this study, healthy young men were included, in part to reduce the risk of injury and hypothetical adverse cardiovascular responses to strenuous exercise under conditions of sleep loss. A second reason to study healthy, young subjects was to reduce inter-individual differences that could have impacted the response to our combined sleep loss-exercise intervention, and thus also have reduced sensitivity; moreover, this made possible the attribution of any exercise-induced changes to differences in sleep duration *per se,* as opposed to a combinatory interplay of sleep restriction and pre-existing morbidity. In total, 16 normal-weight, somatically and psychiatrically healthy young men (mean ± S.E.M., age 23 ± 0.7 years; BMI 23 ± 0.5 kg/m^2^) participated in a randomized and counterbalanced order in two sessions: three nights of normal sleep duration (NS) and three nights of sleep restriction (SR). We chose sample size based on prior interventional sleep restriction studies [[9], [10], [11]] and remained consistent with, or greater than, many acute exercise-focused interventions in healthy individuals [[12], [13], [14]]. The study was approved by the regional Ethics Committee in Uppsala (Dnr 2014/242/1, Sweden) and was conducted following written and orally informed participant consent.

Prior to being enrolled in the study, all participants answered a standardized questionnaire assessing their medical history and current somatic and psychiatric health, followed by a detailed medical interview by a licensed medical doctor (J.C.). All participants were in general good health; none had any sleep condition or other medical conditions, and all were free from medication and nicotine use. Participants did not consume ≥5 units of alcohol per week, and all had a waist circumference <102 cm. None of the participants had carried out any shift work within the previous six months, and none had traveled across more than one time zone within the last four weeks (and no such inter-time zone travel occurred between the two sessions).

Participants were also specifically screened regarding their cardiovascular health status. Participants were excluded if they had any history of cardiovascular diseases (including general heart conditions, hypertension, hypotension, rhythm disorders or thyroid disease). Participants were also asked whether they had a first-degree family history of cardiovascular disease: general heart conditions (0 out of 16 participants), rhythm disorders (0 out of 16 participants) and hypertension (1 out of 16 participants; this participant had one first-degree relative with hypertension, but the participant had normal resting blood pressure, <130/80 mm Hg).

Participants were also screened for hypotension (all had resting systolic blood pressure >90 mm Hg, diastolic blood pressure >60 mm Hg) and hypertension (all had resting systolic blood pressure <130 mm Hg, diastolic blood pressure <90 mm Hg). Finally, all participants showed normal sinus rhythm, a normal P wave, a normal QT interval and a normal QRS complex morphology and duration.

As part of the initial screening, we gathered laboratory values based on participants in a well-rested state: all participants had normal fasting glucose (<6.0 mmol/L) and a normal glucose response to a standardized 75-g glucose tolerance test done from a fasting baseline (2-h glucose values < 7.8 mmol/L). Furthermore, fasting total leukocyte levels (<9 × 10^9^), creatinine (<105 μmol/L), cholesterol (<6.1 mmol/L) and triglyceride (<2.6 mmol/L) levels were all within the normal reference range used by the local hospital clinical chemistry lab.

Only participants who self-reported good sleep were included in the study, as confirmed by general questions about their sleep (“Do you sleep well?“) and by including only those who reported sleeping 7–9 h per night and who scored ≤5 on the Pittsburgh sleep quality index. Prior to participation in the study, participants also filled out a diary over at least seven days to detail their sleep, activity and food consumption habits and to confirm that participants had, for instance, regular sleep/wake habits and consumed three main daily meals.

### 2.2 Study protocol

Prior to the in-lab sessions, participants a) slept in the night to habituate themselves to the environment and screen for a normal polysomnography-based sleep pattern, and b) took part in a submaximal cycling test. Besides habituating the participants, the cycling test allowed the experimenters to calculate the exercise load to achieve 75% VO_2_^maxReserve^.

One week prior and up until the last session, participants were asked to maintain the same sleep, meal and activity schedule. To minimize the risk for carryover effects, a washout period of around 4 weeks elapsed between the first and second intervention periods.

During each experimental session, the participants remained sedentary, and were constantly supervised in designated rooms in the sleep laboratory, across the three in-lab days that preceded the exercise intervention (between 7 a.m. and 10.30 p.m., lights were kept on at 250 lux at eye level). The participants were provided breakfast (8 a.m.), lunch (1 p.m.) and dinner (8 p.m.). These isocaloric meals were individually adjusted in terms of calories (using the Harris–Benedict equation factored 1.2), kept identical across both sessions and always had to be consumed within 20 min of meal onset. In the NS condition, the sleep opportunity each night spanned the period 10.30 p.m. to 7 a.m. In the SR condition, sleep was only possible between 2.45 a.m. and 7 a.m., and lights were kept dim (<6 lux) between 10.30 p.m. and 2.45 a.m. Sleep polysomnography showed that subjects slept on average 4.05 ± 0.03 h per night in the SR condition and 7.95 ± 0.07 h per night in the NS condition. Participants were continuously monitored, and during the two full in-lab days, they were taken on three supervised, slow-paced in-lab walks at 11.15 a.m., 2.30 p.m. and 5 p.m. (60 min total walking time per day).

To investigate possible pre-exercise differences in serum levels of cTnT and NT-proBNP between the sleep conditions, blood was sampled twice in the fasting state: in the evening before and in the morning after the third night (i.e., 7.30 p.m. and 8.30 a.m., respectively). On the last day, serum levels of cTnT and NT-proBNP were determined before (∼10 a.m.) and after (+15, +30, +60, +120, and +240 min) the 30 min of ergometer cycling at vigorous intensity (75% VO_2_^*maxReserve*^). The exercise bout was preceded by a 5-min warmup at a quarter of the subsequent 30-min intensity. During the exercise bout, heart rate was measured using a chest-attached Polar H10 sensor (recording failed for one participant, yielding n = 15 pairs) that was part of the ergometer. On the last day, preceding and following the exercise bout, participants were restricted to cushioned stretchers set to a semi-reclining position and were allowed to drink water.

### 2.3 Biochemical analyses

Serum levels of cTnT were measured with a high-sensitivity assay (lower limit of detection 3 ng/L) using the Cobas 8000 platform (Roche Diagnostics, Rotkreuz, Switzerland). Concentrations of NT-proBNP in serum were also measured on the Cobas 8000 platform. Both assays were analyzed by an experimenter blinded to the study conditions. All other clinical chemistry parameters were measured on a clinical chemistry analyzer (Architect C16000, Abbott Laboratories).

### 2.4 Statistical analyses

Distribution of continuous variables was assessed using the Wilk–Shapiro test for normality. Non-parametric values were first log_2_-transformed prior to analysis. Baseline, pre-exercise (7.30 p.m. and 8.30 a.m.) and exercise-induced differences in serum levels of cTnT and NT-proBNP, between the sleep conditions, were examined with repeated measures ANOVAs, including the within-subjects factors *sleep* (i.e., NS vs. SR) and *time* (reflecting time points). We also investigated possible interaction effects of *sleep* and *time*. Post hoc tests were adjusted for multiple testing using the false discovery rate. All analyses were run in Prism (v.9.3.0). Values are shown as means ± S.E.M.

## 3 Results

### 3.1 Effects of sleep and exercise on serum levels of cTnT or NT-proBNP

Pre-exercise serum levels of cTnT or NT-proBNP did not differ between the SR and NS conditions, as indicated by the circulating levels in the evening before (7.30 p.m.) and morning after (8.30 a.m.) the third in-lab night (repeated measures ANOVA, cTnT: main sleep effect P = 0.54, interaction effect P = 0.88; NT-proBNP, main sleep effect P = 0.58, interaction effect P = 0.52) (Fig. S1). The same analyses did, however, reveal overall (but thus sleep duration-independent) evening-to-morning changes for NT-proBNP but not for cTnT (NT-proBNP: ∼43 ± 21% higher in the evening vs. morning; main *time* effect P = 0.0388) (∼8 ± 4% lower in the evening vs. morning; main *time* effect P = 0.06) (Fig. S1).

**Figure 1:**
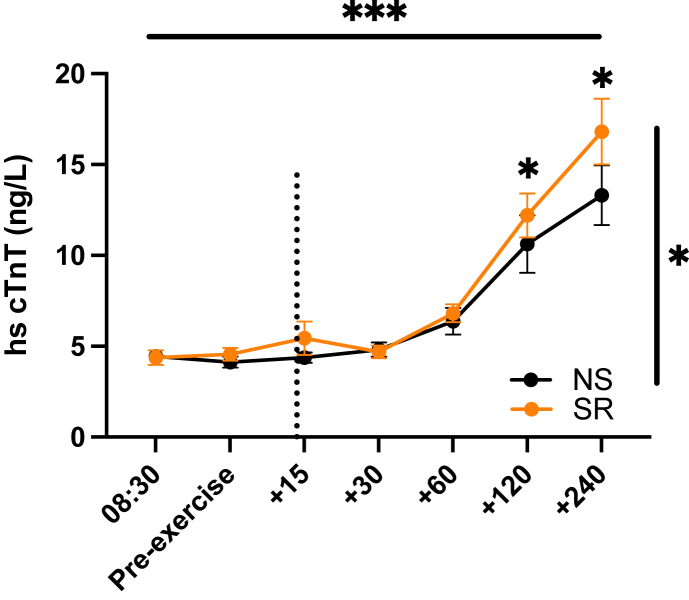
**Recurrent sleep restriction increases circulating levels of troponin T after acute exercise.** Serum levels of cardiac Troponin-T (cTnT) before and after the morning exercise bout, in the normal sleep (NS) and sleep restriction (SR) condition (n = 16 participants). The horizontal line indicates the main time effect; the vertical line the main effect for the sleep condition; individual asterisks indicate differences between sleep conditions at specific (post-hoc-tested) time points. Values shown as mean ± S.E.M. ∗, P < 0.05; ∗∗∗, P < 0.001.

**Figure 2:**
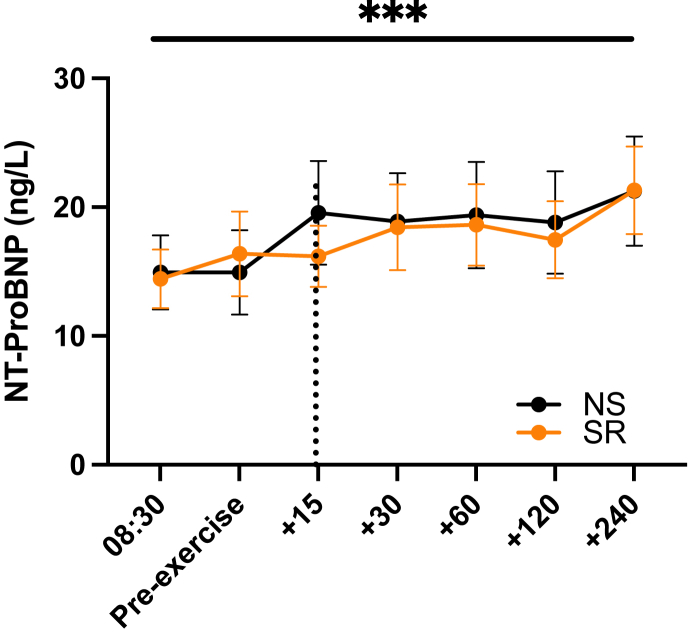
**Recurrent sleep restriction does not increase circulating levels of NT-proBNP after acute exercise.** Serum levels of N-terminal pro b-type natriuretic peptide (NT-proBNP) before and after the morning exercise bout, in the normal sleep (NS) and sleep restriction (SR) condition (n = 16 participants). The horizontal line indicates the main time effect. Values shown as mean ± S.E.M. ∗∗∗, P < 0.001.

In contrast, in response to exercise, serum cTnT levels increased significantly (repeated measures ANOVA main effect for *time*, P < 0.0001), and this surge was significantly greater after SR compared with after NS (main effect for *sleep*, P = 0.0272; *sleep∗time* interaction, P = 0.0778) (Figure 1). Post hoc analyses that were corrected for multiple comparisons revealed cTnT serum levels were significantly higher 120- and 240-min post-exercise (SR/NS ratio, +38 ± 14% for the 120-min time point and +37 ± 14% for the 240-min time point; P = 0.0252 and P = 0.0150, respectively). Furthermore, while serum NT-proBNP levels also increased over time in response to exercise (main effect for *time*, P < 0.0001), this surge did not significantly differ between the sleep condition (main effect for *sleep*, P = 0.80; *sleep∗time* interaction, P = 0.34) (Figure 2).

### 3.2 Heart rate changes during the exercise protocol

As expected, heart rate rose during the exercise protocol (main effect for *time*, P < 0.0001). However, we did not find differences in heart rate between the SR and NS conditions (main effect for *sleep*, P = 0.82; *sleep∗time* interaction, P = 0.92) (Fig. S2).

## 4 Discussion

The new important finding of our study is that a few days of insufficient sleep are associated with a more significant increase in circulating levels of cTnT in response to strenuous aerobic exercise in healthy young men. Based on our present findings, under conditions of chronic short sleep duration, intense physical exercise may be hypothesized to increase the risk of cardiac events, although whether this would apply to otherwise healthy individuals remains unknown. However, higher serum levels of cTnT/cTnI have been associated with subclinical myocardial injury, heart failure and cardiovascular death [15]. Moreover, recent evidence suggests that the cardiac troponin response to exercise predicts higher mortality and risk of cardiovascular events—notably, even after adjusting for cardiovascular risk factors and diseases and baseline troponin values [16].

Exercise-mediated cardiac troponin release may represent a physiologic myocardial stress response. Possibly through its established impact on cellular inflammation and the autonomic nervous system, sleep loss may lower the threshold (i.e., in terms of exercise intensity or duration) at which an increased level of myocardial stress occurs. However, while data exist to indicate that even partial sleep restriction can alter the autonomic nervous system tone—as evidenced by reduced heart rate variability (HRV) [11]— evidence also exists to indicate no change to HRV in response to high-intensity interval exercise under conditions of partial sleep deprivation [17]. Sleep loss has been shown to result in tissue-specific circadian disruption [18,19]. Thus, sleep loss could misalign the molecular clock within cardiomyocytes, which controls numerous key homeostatic processes such as metabolic substrate utilization [20]. Consequently, under such conditions these cells may become more vulnerable to physiological stressors such as high-intensity exercise. Indeed, a prior study has demonstrated that the circadian clock regulates the sensitivity to perioperative damage and that this difference is reflected clinically by a morning vs. afternoon difference in release of perioperative cTnT [21].

One strength of our study is that we used a highly standardized in-lab paradigm where participants were continuously monitored and in which activity, meal and light exposure were all standardized. Combined with our within-subject design, this ensured that the observed differences should be attributable to our sleep intervention. We also assessed levels of cTnT and NT-proBNP under evening and morning resting conditions, but only observed sleep loss-mediated differences in response to a physiologic stressor, i.e., a bout of exercise. Another strength of our study is that all participants had undergone a detailed cardiovascular screening, highlighting that the observed effects occur in young individuals who were in good cardiovascular and general health.

There remain some important considerations when interpreting our findings. Sleep loss can promote inflammation and disrupt the regulation of the autonomic nervous system, including heart rate regulation [11,22]. How these parameters interact with our findings remains to be explored. For instance, certain studies have observed sleep loss-induced differences in heart rate variability following exercise. Whether such electrophysiological alterations may have contributed to our present findings remains unresolved.

Herein, partly to reduce inter-individual variability and thereby increase the chance to detect a difference, we studied a single and quite vigorous exercise intensity and only included healthy young adult men, and only those who habitually reported sleeping the recommended number of hours per night. Our results may therefore not generalize to women, people with cardiovascular disease, or to other age groups. Even in our set of participants who had been extensively screened for cardiovascular disease, we did note that the exercise-induced troponin release exhibited substantial inter-individual variability, consistent with data from prior acute exercise interventions [14]. It is therefore possible that some individuals are more resilient than others to the effects observed herein. This aspect may be possible to explore in interventions that employ larger sample sizes as well as multiple exercise intensities. Indeed, while troponin release may be more pronounced at an even greater exercise intensity [12], our intervention does represent an exercise intensity that is commonly experienced over a similar duration in those who engage in recreational sports or exercise training. As such, our findings may bear some clinical relevance, and may be relevant to consider when evaluating patients both in the context of recreational exercise training, as well as in competitive elite training.

In our study, we utilized a recurrent sleep loss paradigm that, while mirroring recurrent sleep loss that occurs in everyday life, may have been more pronounced than that experienced by many individuals. It is therefore possible that our findings would be different in response to other types of sleep loss, such as overnight wakefulness (e.g., experienced by many shift workers [23]), or following more subtle, but more prolonged, chronic sleep restriction. A related, important aspect will be to examine whether intermittent (ad libitum) recovery sleep, in between periods of recurrent sleep restriction, can fully reset the adverse sleep loss-mediated impact on exercise-induced troponin release [24].

Another important aspect will be to investigate whether acute exercise induces a similar troponin response—as we observed in this study—in those suffering from chronic sleep disruption, e.g., in those with insomnia or obstructive sleep apnea [25]. Finally, results from a recent large-scale prospective study found that self-reported levels of physical exercise may offset the overall harmful effects of insufficient sleep on cardiovascular health [26]; however, whether regular bouts of strenuous physical activity, as applied in our study, benefit cardiovascular health among habitual short sleepers remains unclear.

Many individuals must, or choose to, carry out intense exercise under conditions of disrupted sleep/wake cycles, for instance, forgoing sleep to workout. Whether frequent exercise under conditions of chronically disrupted sleep can promote long-term damage to cardiomyocytes, potentially primarily in older or other individuals who already suffer from—or are at risk of—cardiovascular disease, remains to be determined. Our results further highlight the importance of considering sleep when evaluating cardiovascular biomarkers, especially in the context of exercise and in young individuals.

### Sources of funding

This work was supported by the Swedish Society for Medical Research (SSMF), the Swedish Research Council and the following foundations: Diabetesfonden (2019-489), Diabetes Wellness Sverige (25-2231), Göran Gustafsson (2019-1941), Magnus Bergvall (2019–03522), Mats Kleberg (2018-00081), NovoNordisk (NNF19OC0056694), Selander, the Swedish Cancer Foundation (19 0269 Pj) and the Swedish Brain Foundation (FO2020-0334). The study sponsor/funding agencies were not involved in the design of the study or in the collection, analysis and interpretation of data, nor were they involved in writing the report; finally, the sponsor/funding agencies did not impose any restrictions regarding the publication of the report.

## Author contributions

J.C. came up with the study idea and designed the study with input from C.B.; J.C. wrote the protocol and conducted the experiments; T.M., F.S. and J.C. conducted the analyses; T.M., F.S., C.B., T.O. and J.C. interpreted the data; J.C. wrote the original manuscript draft. All authors contributed to review and editing and approving the final version. All authors take responsibility for the integrity of the data and the accuracy of the data analysis.

## Data availability statement

The data underlying this article will be shared upon reasonable request to qualified researchers.
