# Supplementary figures and images for: Effects of curtailed sleep on cardiac stress biomarkers following high-intensity exercise
Source: Mol Metab. 2022 Jan 26;58:101445. doi: 10.1016/j.molmet.2022.101445 (PMC8885606; doi:10.1016/j.molmet.2022.101445)

**A**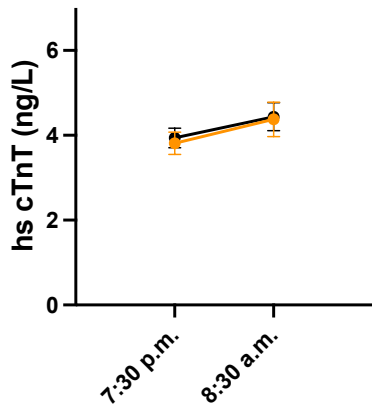**B**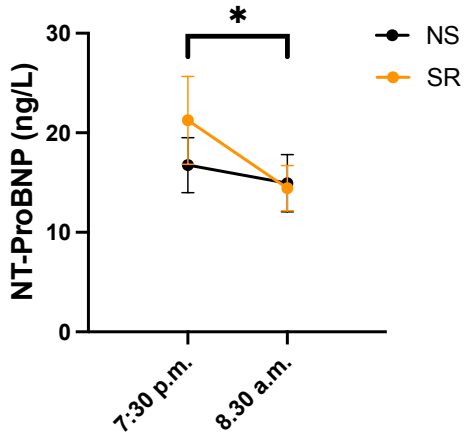

Supplement: Multimedia component 1 [file mmc1.pdf]

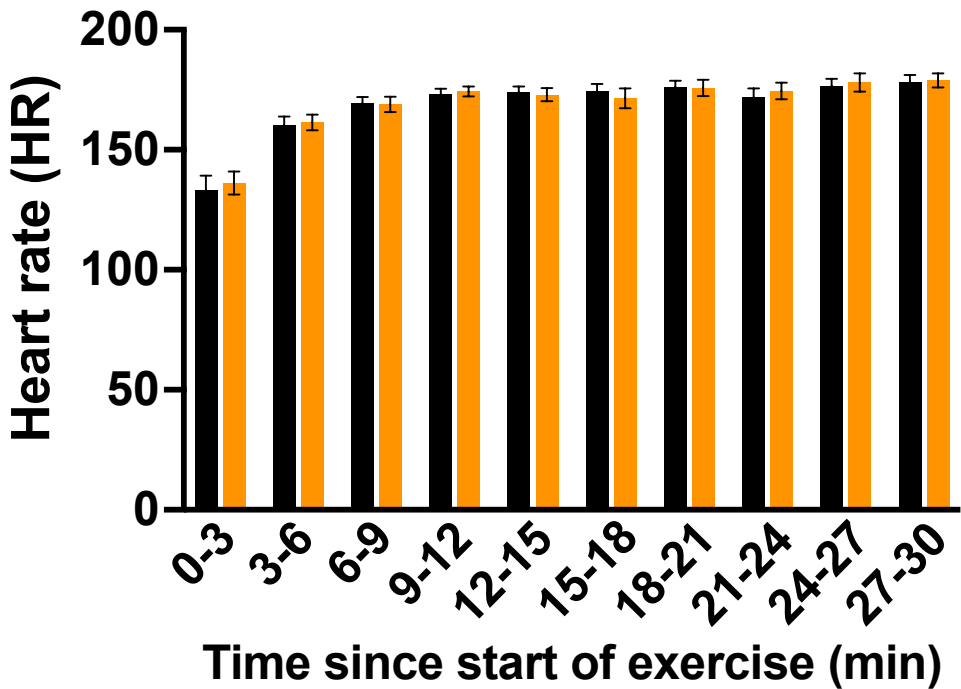

Supplement: Multimedia component 2 [file mmc2.pdf]
